# Pollinator Species Identity, Behavioural Ecology and Life History Traits Shape Epibiont Bacterial Diversity

**DOI:** 10.1007/s00248-026-02845-9

**Published:** 2026-07-23

**Authors:** V. Aiko Lignon, E. Eirian Jones, Manpreet K. Dhami, Clive Kaiser, Flore Mas

**Affiliations:** 1 Department of Pest Management and Conservation, Faculty of Agriculture and Life Sciences, Lincoln University, Lincoln, New Zealand; 2 Bioeconomy Science Institute – Maiangi Taiao, PO Box 69040, Lincoln, 7640 New Zealand; 3 Department of Agricultural Sciences, Faculty of Agriculture and Life Sciences, Lincoln University, Lincoln, New Zealand; 4 Bioeconomy Science Institute – Maiangi Taiao, Lincoln Science Centre, 74 Gerald Street, Lincoln, 7608 New Zealand

## Abstract

Pollinators interact with flowers partly through direct surface contact, yet the bacterial communities associated with their external surfaces (epibionts) remain poorly understood compared with their gut microbiomes. This study characterised the epibiont bacterial communities of four pollinator species associated with northern highbush blueberry (*Vaccinium corymbosum* ‘Duke’) in New Zealand: bumble bee (*Bombus terrestris*), honey bee (*Apis mellifera)*, drone fly (*Eristalis tenax*), and a New Zealand native bee (*Lasioglossum sordidum*). Using 16 S rRNA gene sequencing, we detected distinct epibiont communities among pollinator species, with significant differences in alpha and beta diversity. Bumble bees consistently exhibited specialised and low‑diversity communities, whereas drone flies showed the highest bacterial diversity. Several pollinator gut‑associated core bacterial genera, including *Gilliamella*, *Lactobacillus*, and *Snodgrassella*, were detected across most species, representing the first reports of *Snodgrassella* on *E. tenax* and of *Lactobacillus* and *Gilliamella* on *L. sordidum*. Environmentally acquired taxa dominated the epibiont communities, consistent with pollinator exposure to floral and habitat‑specific bacterial sources. Differential abundance analysis identified species‑specific bacterial signatures and highlighted how pollinator identity, behavioural ecology, and life history traits shape epibiont composition.

**Supplementary Information:**

The online version contains supplementary material available at https://doi.org/10.1007/s00248-026-02845-9.

## Introduction

Pollination is a fundamental ecological process necessary for plant reproduction, especially for about a third of the crops that are produced around the world [1]. While the necessity and effectiveness of pollinators in transferring pollen is well understood, the potential additional transfer of microbial communities influencing plant fitness, especially bacteria associated with pollinator body surfaces, remains under-characterised in this tripartite interaction between plants, pollinators and microbes [2]. Certain pathogenic fungi, including *Botrytis cinerea*, are known to be transmitted by insects and can adversely impact plant immunity and reproductive success. Similarly, yeast, such as *Metschnikowia reukaufii*, has been shown to influence floral volatiles and, consequently, pollinator visits [2]. Yet, despite this growing recognition of microbial influences on plant-pollinator interactions, the role of bacteria in this tripartite interaction remains comparatively underexplored.

Most pollinator microbiome research has focused on the gut, especially in eusocial bees such as honey bees (*Apis mellifera*) and bumble bees (*Bombus* species), where a conserved core bacterial community that supports digestion, pathogen defence, and colony health [3–6]. Additionally, relatively less attention has been given to other pollinators, such as solitary bees and flies [7, 8]. In contrast, far fewer studies have examined bacterial communities associated with the external surface of pollinators, here referred to as the epibiont microbiome, defined here as the assemblage of “organisms living on the surface of another living organism” [9]. Existing studies of pollinator epibiont microbiomes are limited and have been largely focused on honey bees (*A. mellifera*) [10–12], leaving epibiont communities across diverse pollinator species comparatively under-characterised.

Unlike the internally regulated gut microbiome, epibiont communities occupy an interface between pollinators, plants, and the environment. These surface-associated bacterial assemblages are shaped by environmental exposure and direct contact with floral tissues, and they are therefore positioned to interact with the bacterial communities of flowers, pollen and stigmas [13–15]. During pollination, bacteria can be exchanged bi-directionally between insects and plants at the floral interface [2, 14, 16] positioning pollinators as mobile bacterial vectors. Pollinator species identity and behavioural ecology may influence the taxa they acquire and transmit [2], while flowers serve as local bacterial reservoirs [17]. This is consistent with experimental studies in solitary bees demonstrating life‑stage‑specific microbiome acquisition and transmission [18].

Despite their potential importance in mediated plant-pollinator-microbe interactions, epibiont bacterial communities remain poorly characterised across many pollinator groups, particularly solitary bees and flies. Understanding how epibiont bacterial communities differ among pollinator species with contrasting life histories and behavioural ecology is critical for assessing their potential roles in influencing the bacterial succession and potential health outcomes of the pollinator and/or plant [16]. Unlike the internally regulated gut microbiome, epibiont communities constitute a distinct and largely transient assemblage shaped by environmental exposure and direct contact with floral surfaces, capturing short-term bacterial acquisition and transmission processes central to plant–pollinator interactions [13, 14, 16].

Even though bacterial exchange occurs during pollination, there remains a substantial gap in our understanding of the epibiont bacterial microbiome of pollinators. Pollinators associated with highbush blueberries (*Vaccinium corymbosum*) provide an ideal system for examining these dynamics. Globally, blueberry production involves a diverse assemblage of pollinators, including honey bees (*A. mellifera*), bumble bees (various *Bombus* spp.), wild/native bees and flies [19–22]. In New Zealand, where blueberries are an introduced crop, commercial pollination is dominated by managed honey bees [23], although bumble bees are more efficient pollinators on a per-bee basis due to their ability to buzz-pollinate blueberry flowers [24]. The New Zealand blueberry pollination system offers a useful contrast, comprising managed eusocial pollinators that likely experience relatively structured, hive‑associated bacterial reservoirs, alongside a small number of wild pollinator species that encounter a more variable and diverse environmental bacterial sources. These differences in behavioural ecology provide a way to examine how pollinator identity, behavioural ecology and life history traits shape epibiont community assembly. Together, these contrasts highlight the need to understand how pollinator species with contrasting ecologies differ in their potential exposure to environmental and flower‑associated bacteria, including those encountered during pollination. Accordingly, this study takes an exploratory, descriptive approach to characterising epibiont bacterial communities of four pollinator species following shared exposure to a blueberry pollination environment, to compare community composition across pollinators with differing life histories and exposure pathways.

## Materials and Methods

### Plant Material

The experiment was conducted in October 2022 and 2023 at Bioeconomy Science Institute – Plant and Food Research (BSI-PFR), Lincoln, New Zealand. Approximately two-year-old northern highbush blueberry (*Vaccinium corymbosum* ‘Duke’) plants were sourced from Southern Woods Nursery, Rolleston, New Zealand, and maintained in a shade house on campus in acidic potting mix consisting of bark, peat and pumice. Plants were watered as required by hand using a hose.

BugDorm^®^ (W60 x D60 x H120 cm) insect rearing cages were positioned on a grassy area on the campus. Each cage contained a single blueberry plant and was spaced 100 × 50 cm in 2022 or 80 × 80 cm in 2023. Cages were randomly assigned a pollination species.

### Pollinator Species

In 2022, the pollinator species included bumble bees (*Bombus terrestris*), drone flies (*Eristalis tenax*), and honey bees (*Apis mellifera*). In 2023, pollinator species consisted of bumble bees (*B. terrestris*) and a New Zealand native bee (*Lasioglossum sordidum*). Eusocial bees (bumble bee and honey bee) were sourced from commercial bumble bee hives (Biobees Ltd, Hastings, New Zealand) and from managed honey bee hives located at the BSI-PFR, Lincoln campus. Both bumble bees and honey bees were collected using 50 mL sterile conical tubes by intercepting individual foragers as they left the hive or retrieving them upon return. Drone flies were reared from wild-collected pupae material following the protocol established by Nicholas et al. [25] to get a constant supply of drone flies. Emerging adults were maintained on honey and water inside BugDorm^®^ (W30 × D30 × H30 cm) insect-rearing cages. Individuals were collected by gently scooping them from the inner cage surface with 50 mL sterile tubes. *L. sordidum* were gathered from an aggregation site of ground nests near Springston, Canterbury, New Zealand (− 43.641015, 172.388961) by placing 120 mL sterile containers upside down over nest entrances as bees entered or exited.

Pollinators (four per cage) were introduced to caged blueberry plants and left to forage freely within cages for 6 h in 2022 and 72 h in 2023 to standardise exposure to the blueberry pollination environment before individual pollinator sample collection. Pollinator activity was visually observed during the exposure period, and individuals were seen visiting blueberry flowers within the cages. This design ensured that individuals had recent access to blueberry flowers while maintaining natural foraging behaviour. As pollinators were collected from the field before caging, the epibiont communities likely reflect a combination of pre-capture environmental exposure and subsequent exposure within the cages, which cannot be disentangled in this study. Pollinators were not sampled directly from flowers, and the epibiont microbiome is therefore interpreted as reflecting recent environmental and floral contact rather than exclusive flower‑specific acquisition.

At least nine individuals were collected for each pollinator species, with some individuals lost due to stress-induced mortality in the cages, particularly among honey bees. All samples were transported to the laboratory on ice, euthanised at − 20 °C, and stored until processing. Only bumble bees were sampled in both 2022 and 2023, therefore, allowing descriptive comparisons of patterns across sampling years for this species. Data from honey bees, drone flies, and *L. sordidum* represent single-year samples and were not used to infer temporal or seasonal patterns.

### DNA Extraction and Bacterial 16S rRNA Gene Amplicon Sequencing

To collect and analyse the bacterial epibiont of each pollinator species, the external surface of each insect was washed. In 2022, to wash the external surface of the pollinators, each bumble bee was placed in an individual 5 mL sterile Eppendorf tube, whereas each drone fly, honey bee, and *L. sordidum* (in 2023) was placed in a 1.7 mL sterile Eppendorf tube. All tubes contained 1 mL of ultrapure MilliQ water. In 2023, the water volume for bumble bees was increased to 2 mL to ensure total submersion. Tubes were vortexed for 10 s and then sonicated for 2 min using an ultrasonic frequency of 37 kHz at 30 °C (Sono Swiss SW 12 H PRESET, Switzerland), after which the insect was removed and the remaining liquid, referred to as the external surface wash, was stored at − 20 °C until further processing. DNA extractions were performed using the Macherey‑Nagel NucleoSpin^®^ 96 Plant II kit (MACHEREY‑NAGEL GmbH & Co. KG, Düren, Germany) following the manufacturer’s vacuum‑filtration protocol on a Janus4 Liquid Handler Workstation (PerkinElmer, CT, USA), with 100 µL of each external surface wash used as input, Buffer PL1 as the lysis reagent, and a final DNA elution volume of 100 µL.

Following DNA extraction, a two-step metabarcoding amplification approach was performed. In the first polymerase chain reaction (PCR) step, the V4 region of the bacterial 16S rRNA gene was amplified using primers 515F/806R [26]. Reactions (15 µL) contained 2 µL of template DNA and were run under the following conditions: 95 °C for 2 min; 35 cycles of 95 °C for 20 s, 56 °C for 20 s, and 72 °C for 30 s; with a final extension at 72 °C for 2 min. Each PCR run included a no-template (MilliQ water) negative control and a mock community positive control to monitor contamination and amplification performance. PCR success was confirmed on a 2% agarose gel. Products were purified using Omega Bio-Tek Mag-Bind^®^ TotalPure Next-Generation Sequence beads (Omega Bio-Tek, Inc., Georgia, USA), following the manufacturer’s instructions, and used as a template for the second PCR with dual‑indexed Fusion primers. Second‑step reactions (18 µL) contained 1.8 µL of cleaned product and were cycled at 95 °C for 2 min; 8 cycles of 95 °C for 20 s, 50 °C for 20 s, and 72 °C for 30 s; and 72 °C for 2 min. Amplicons were again magnetic bead‑purified to remove primer dimers.

Library concentrations were quantified using Qubit^®^ fluorometry (Invitrogen dsDNA HS Assay Kit, Oregon, USA) and quantitative PCR (Invitrogen™ Collibri™ Library Quantification Kit, Lithuania), and fragment sizes were assessed with a High Sensitivity DNA Assay on the 2100 Bioanalyzer (Agilent, California, USA). Final pooled libraries were diluted to 6 nM and sequenced on an Illumina MiSeq platform (2 × 250 bp) at the Ecological Genetics Laboratory, Bioeconomy Science Institute – Manaaki Whenua Landcare Research (BSI-MWLR), Auckland campus, New Zealand.

### Bioinformatics and Statistical Analysis

Individual pollinator samples from 2022 and 2023 were sequenced on separate Illumina MiSeq sequencing chips, so to avoid introducing potential batch effects, the data from each year were analysed separately. The raw BCL files generated by the Illumina MiSeq platform were converted to FASTQ format using bcl2fastq2 (version 2.20). Demultiplexing of sequences was then performed using Claident (version 0.2.2018.05.29) [27]. Sequence processing and analysis were carried out using the DADA2 package (version 1.26.0) following the pipeline version 1.16 within RStudio (version 2022.7.1.554) [28] on the Research and Education Advanced Network New Zealand Ltd (REANNZ) OnDemand environment [29]. Forward and reverse reads were first trimmed to remove primer sequences, truncated based on quality profiles (Supplementary Fig. 1), merged using the default parameters, chimeras removed, and the resulting amplicon sequence variants (ASVs) were filtered by length (Supplementary Table 1). Taxonomic assignment was conducted using naïve Bayes classifiers with the SILVA 16S rRNA reference database (version 132) [30]. A de novo phylogenetic tree was constructed from ASV sequences. ASV sequences were aligned using DECIPHER (version 2.26.0), and a neighbour-joining tree was inferred from a maximum‑likelihood distance matrix. The tree was subsequently optimised under a GTR model using phangorn and rooted before downstream analysis.

Statistical analyses and graphical outputs were performed in R (version 4.2.1) [31] using RStudio, utilising the following R packages: phyloseq (version 1.40.0), decontam (version 1.18.0), metagenomeSeq (version 1.40.0), vegan (version 2.6.6.1), ggplot2 (version 3.5.1), UpSetR (version 1.4.0) and DESeq2 (version 1.38.3). ASV and taxonomy tables were combined with sample metadata and a phylogenetic tree to generate phyloseq objects; samples that did not meet sequencing or upstream quality-control criteria were excluded at this stage. Contaminant ASVs (including mitochondrial and chloroplast sequences) were identified and removed using decontam based on PCR negative controls, and samples with fewer than 100 reads (based on rarefaction curves; Supplementary Fig. 2) were excluded before downstream analyses. Data were normalised using cumulative sum scaling (CSS) using metagenomeSeq. Alpha diversity was quantified using the Shannon diversity index, and beta diversity was evaluated with non‑metric multidimensional scaling (NMDS) ordinations based on weighted UniFrac distances to visualise community patterns. In addition, beta dispersion was utilised to assess the homogeneity of multivariate dispersion among pollinator species using Bray–Curtis dissimilarities and using ANOVA to test for significant differences in dispersion (Supplementary Fig. 3). Differences in the bacterial community composition among pollinators were assessed using permutational multivariate analysis of variance (PERMANOVA). Taxa were aggregated up to the genus level for visualisation. Genera shared among or unique to pollinator species were identified by generating UpSet plots. DESeq2 was used to assess differential abundance between pollinator species combinations each year, using poscounts‑normalised data, removing zero‑count taxa, and applying an adjusted *p*‑value threshold of 0.01 to generate log₂ fold change plots. Genera with an ASV frequency of five or greater were categorised as gut‑associated, gut/environmental, or environmentally acquired. Classification was based on literature-derived evidence from published gut microbiome studies of the focal pollinator species used in this study (Supplementary Table 2). Genera with documented, consistent associations with pollinator guts were classified as gut-associated; those widely reported in both gut and environmental microbiomes were classified as gut/environmental (based on published floral/nectar microbiome studies of blueberries (*Vaccinium* spp.) – Supplementary Table 3); and genera lacking evidence of gut association in these studies were conservatively classified as environmentally acquired, reflecting their predominant association with plant, soil, water, and other environmental sources.

## Results

After quality control filtering, including merging and chimera removal, a total of 1.8 million paired reads were obtained for external surface wash samples from pollinators in 2022, and 1.6 million paired reads in 2023. Statistical analysis of the amplified 16S rRNA (V4 region) data comprised a subset of 59 samples in 2022 (27 bumble bees, 24 drone flies, 8 honey bees) representing 949 ASVs, and 27 samples in 2023 (18 bumble bees, 9 *L. sordidum*) representing 437 ASVs. As pollinator species differed between years, observations across years were limited to bumble bees, which were sampled repeatedly.

The PERMANOVA using Bray–Curtis dissimilarities revealed significant differences in bacterial community composition among the pollinator species (R² = 0.26, *F₂*,*₅₆* = 9.78, *p* = 0.001) and again in 2023 (R² = 0.22, *F₁*,*₂₅* = 7.03, *p* = 0.001) (Supplementary Table 4).

**Fig. 1:**
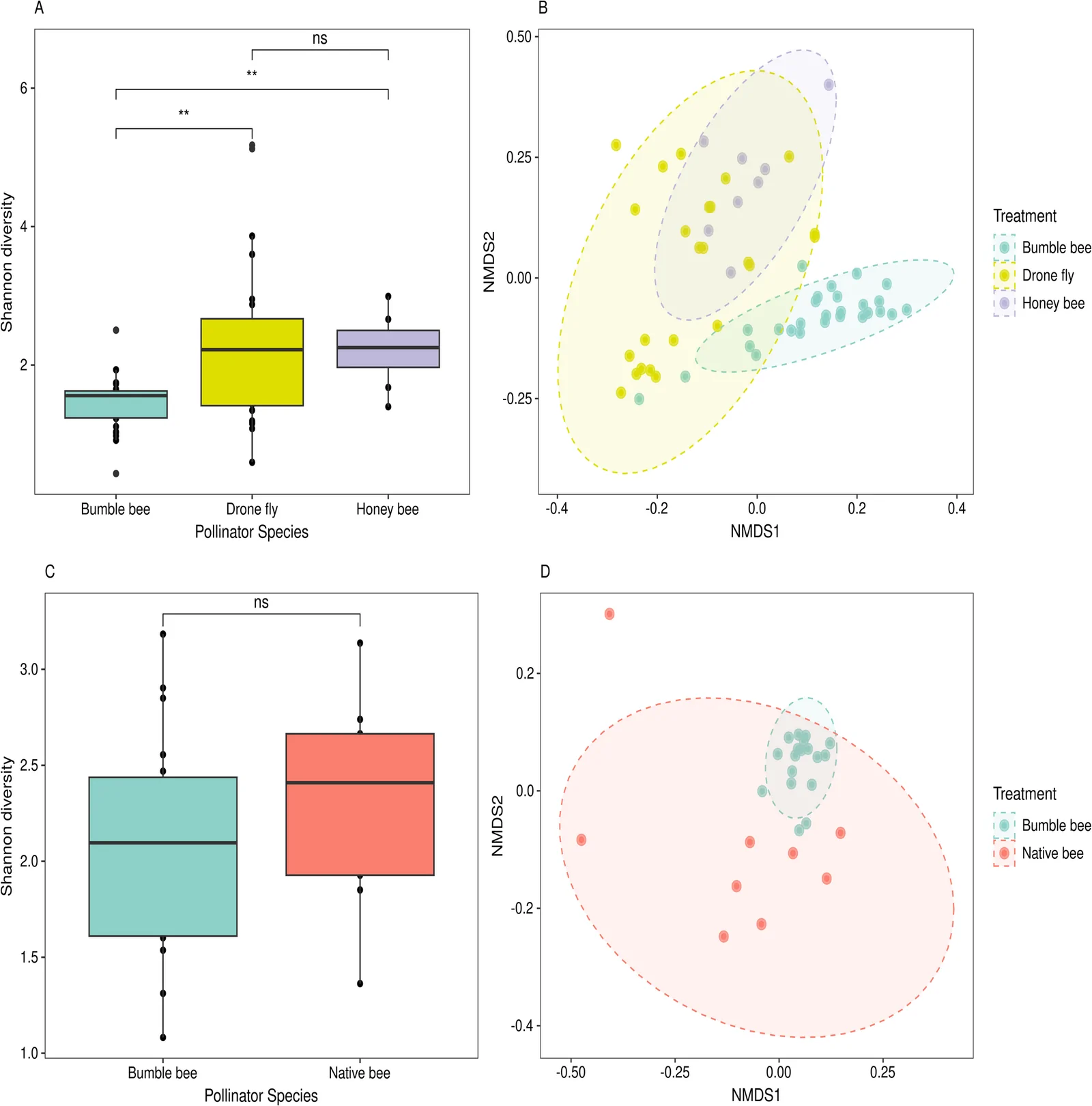
Alpha diversity (Shannon index) of bacterial communities associated with pollinator species (**A**) in 2022 for bumble bee (*Bombus terrestris)*, drone fly (*Eristalis tenax*) and honey bee (*Apis mellifera*), and C) in 2023 for bumble bee (*B. terrestris*) and native bee (*Lasioglossum sordidum*). Points represent individual samples; boxplots summarise within‑species variation. ** = *p* ≤ 0.01; ns = non‑significant. Non‑metric multidimensional scaling (NMDS) of weighted UniFrac distances for (**B**) 2022 and D) 2023. Points represent individual samples coloured by pollinator species; ellipses show 95% confidence intervals

Alpha diversity, measured as the Shannon diversity index, varied across pollinator species in 2022 (Fig. 1A). Bumble bees exhibited significantly lower alpha diversity compared to both drone flies (*p* = 0.002) and honey bees (*p* = 0.003), while there was no significant difference between drone flies and honey bees (*p* = 0.82). In 2023 (Fig. 1C), alpha diversity did not differ between the bumble bees and *L. sordidum* (*p* = 0.32). NMDS ordination supported these patterns (Fig. 1B&D). In 2022, bumble bee and honey bee samples clustered distinctly, while drone fly samples showed higher within‑group variability and partial overlap with both bee species. In 2023, bumble bees clustered closely, while *L. sordidum* displayed broader within-group variation and a slight overlap with bumble bees, indicating higher individual variability in the bacterial community.

**Fig. 2:**
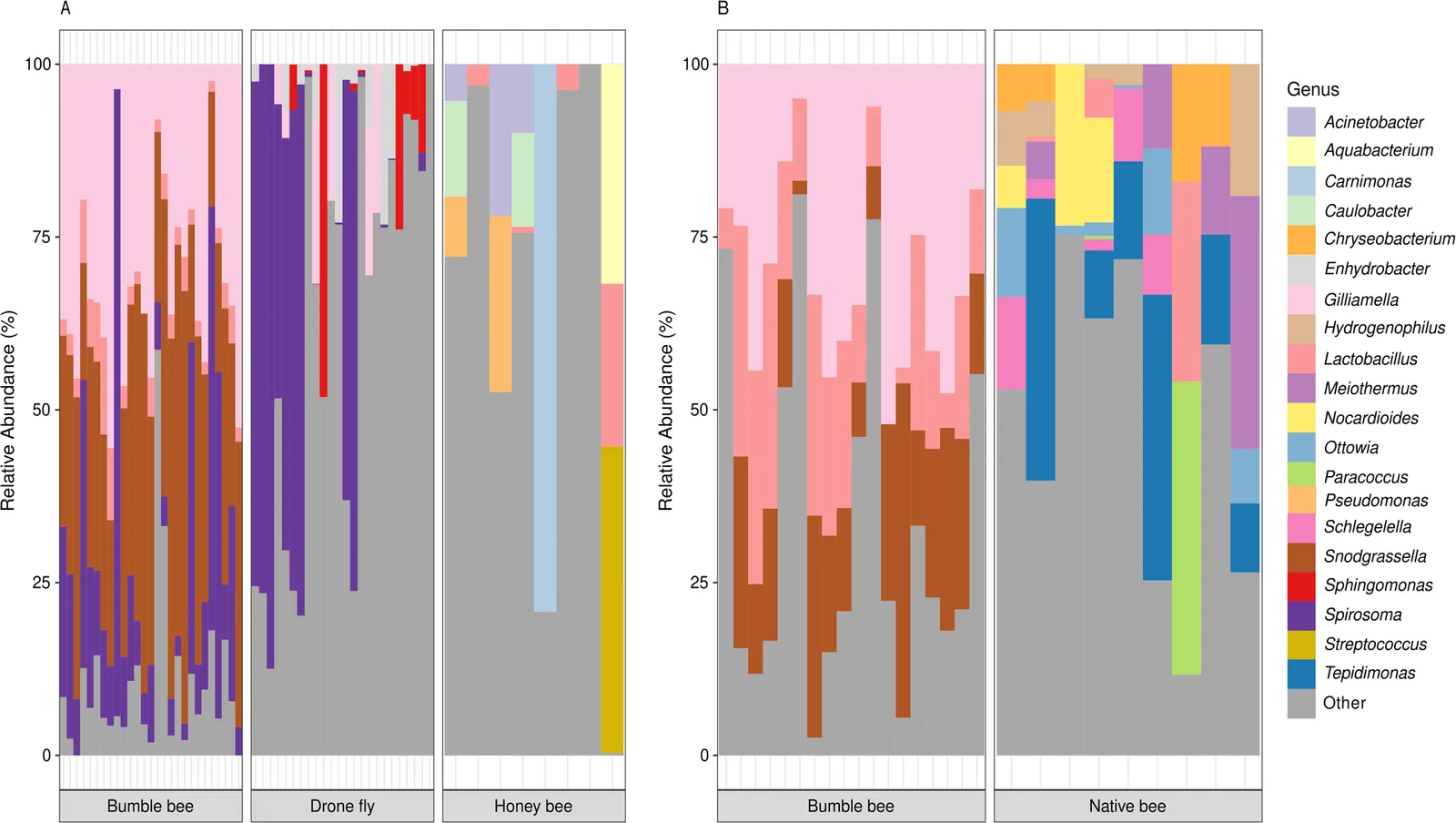
Relative abundance of bacterial genera (with mean ≥ 3%) for each different pollinator species (**A**) in 2022 for bumble bee (*Bombus terrestris*), drone fly (*Eristalis tenax*) and honey bee (*Apis mellifera*) and (**B**) in 2023 for bumble bee (*B. terrestris*) and native bee (*Lasioglossum sordidum*). Each bar represents the bacterial community composition of an individual sample within each pollinator species. Colours indicate different bacterial genera

Epibiont bacterial community at the phylum level for the different pollinator species was dominated by *Proteobacteria*, followed by varying proportions of *Firmicutes*,* Bacteroidetes*, and *Actinobacteria* in both years. Overall, the ASV reads were assigned to 278 bacterial genera in 2022 and 177 bacterial genera in 2023. Relative abundance patterns of bacterial genera varied among pollinator species and individuals (Fig. 2). In 2022 (Fig. 2A), bumble bee samples showed comparatively consistent epibiont communities dominated by a small number of genera, particularly *Gilliamella*,* Lactobacillus*, *Snodgrassella*, and *Spirosoma*, whereas drone flies exhibited high inter‑individual variability with a diverse assemblage of bacterial genera across samples. Honey bee samples also showed significant individual variation, with different genera dominating different individuals and a large contribution from low‑abundance taxa grouped as “other”. In 2023 (Fig. 2B), bumble bees again displayed relatively similar epibiont community profiles in comparison to 2022 samples, with the exclusion of *Spirosoma*. *L. sordidum* exhibited high variability in genus‑level composition among individual samples. Overall, in 2022, the ASVs for bumble bee were assigned to 135 genera, 209 for drone fly and 86 for honey bee, whereas in 2023, it was 143 for bumble bee and 70 for *L. sordidum*.

**Fig. 3:**
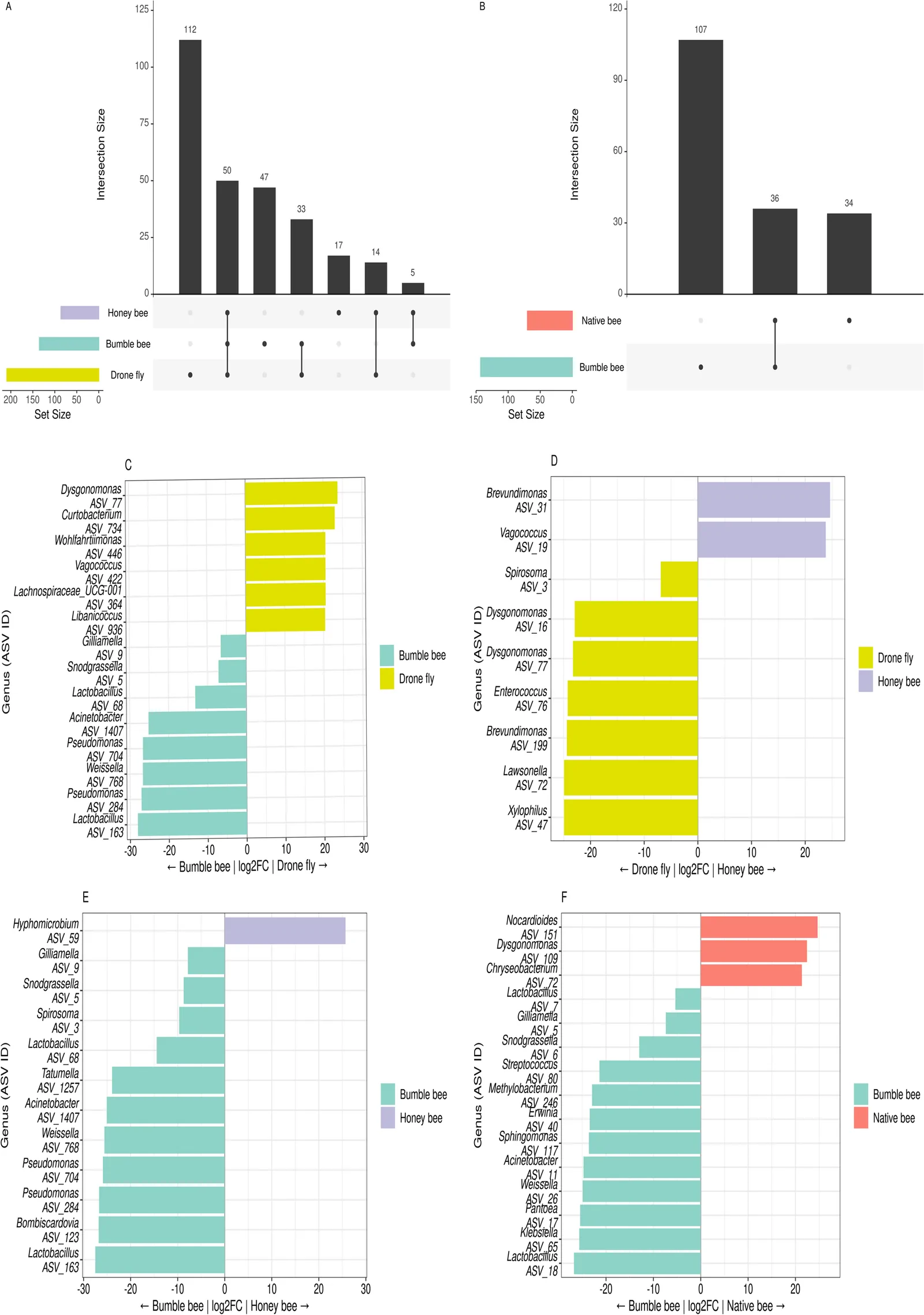
UpSet plots showing shared and unique bacterial genera among different pollinator species (**A**) in 2022 for bumble bees (*Bombus terrestris*), drone flies (*Eristalis tenax*), and honey bees (*Apis mellifera*) and (**B**) in 2023 for bumble bees (*B. terrestris*) and native bees (*Lasioglossum sordidum*). DESeq2-based differential abundance analysis identified amplicon sequence variants significantly enriched in (**C**) drone flies versus bumble bees (2022), (**D**) honey bees versus drone flies (2022), (**E**) honey bees versus bumble bees (2022), and (**F**) native bees versus bumble bees (2023). Bars represent log2 fold changes; only ASVs with FDR-adjusted *p* < 0.05 are shown. Colours indicate pollinator species

To determine which genera were predominantly shared among epibionts of each pollinator species, UpSet graphs were constructed. In 2022, the number of shared genera between the pollinator species was 50 between bumble bees, drone flies and honey bees, with an additional 33 genera between bumble bees and drone flies, 14 between drone flies and honey bees, and 5 between bumble bees and honey bees (Fig. 3A). Of the shared genera, significant ones included *Spirosoma*,* Snodgrassella*,* Gilliamella*,* Dysgonomonas*, and *Vagococcus*. There were 36 shared genera between bumble bees and *L. sordidum* in 2023 (Fig. 3B), including *Gilliamella*,* Snodgrassella*,* Lactobacillus*,* Acinetobacter*, and *Meiothermus*.

In 2022, several ASVs were repeatedly identified as significantly enriched within specific pollinator species across multiple differential abundance contrasts using DESeq2. In drone fly samples, the ASV classified as *Dysgonomas* (ASV_77) was significantly enriched relative to both bumble bee and honey bee samples (Fig. 3C & D). Bumble bees showed enrichment of multiple ASVs, including *Lactobacillus* (ASV_163, ASV_68), *Pseudomonas* (ASV_264, ASV_704), *Weissella* (ASV_768), *Acinetobacter* (ASV_1407), *Snodgrassella* (ASV_5), and *Gilliamella* (ASV_9), when compared with drone fly and honey bee samples (Fig. 3C & E). In contrast, honey bees exhibited relatively few enriched ASVs, with two detected in comparisons with drone flies and one in comparisons with bumble bees (Fig. 3D & E). Consistent with these patterns, differential-abundance analysis for 2023 again identified ASVs classified as *Lactobacillus*, *Gilliamella*, and *Snodgrassella* as significantly enriched in bumble bees, whereas ASVs classified as *Nocardioides*, *Dysgonomas*, and *Chryseobacterium* were enriched in *L. sordidum* (Fig. 3F).

Among the most frequently detected genera with an ASV frequency of five or greater in pollinator samples in 2022, 10.0% were classified as gut‑associated, 7.5% as gut/environmental, and 82.5% as environmentally acquired (Supplementary Table 5). Similar proportions were observed in 2023, with 12.5% identified as gut‑associated, 8.3% as gut/environmental, and 79.2% as environmentally acquired (Supplementary Table 6). The ranking of genera is based on ASV detection frequency across the dataset and reflects prevalence rather than uniform abundance across pollinator species. Across both years, the gut‑associated genus *Lactobacillus* was the highest‑ranked taxon detected in the epibiont bacterial communities of pollinators (Table 1). Other gut‑associated genera detected in both years included *Gilliamella* and *Snodgrassella*, while *Bombiscardovia* was detected only in 2022. *Pseudomonas* and *Acinetobacter* were the only additional genera with reported associations with gut taxa and were classified as gut/environmentally acquired. The most frequently detected environmentally associated genus was *Spirosoma* in 2022 and *Tepidimona*s in 2023. The only environmentally associated genus detected among the top 12 taxa in both years was *Sphingomonas*. Among genera with ASV frequencies of five or greater detected in both years, 43 were environmentally associated, 4 were gut‑associated, and 3 were gut/environmentally associated (Supplementary Tables 5 and 6).

**Table 1:** Top 12 most frequently detected genera based on ASV frequency (mean relative abundance > 1%) in the epibiont bacterial communities of each pollinator species in 2022 for bumble bees (*Bombus terrestris*), drone flies (*Eristalis tenax*), and honey bees (*Apis mellifera*) and in 2023 for bumble bees (*B. terrestris*) and native bees (*Lasioglossum sordidum*). Each genus is classified as gut‑associated, environmental, or gut/environmental to indicate the potential source from which it was acquired and the pollinator species in which it was detected

Year	Genus	ASV Frequency in Samples	Classification	Pollinator Species Found in
2022	*Lactobacillus*	60	Gut-associated	Bumble bee, Drone fly, Honey bee
	*Gilliamella*	50	Gut-associated	Bumble bee, Drone fly, Honey bee
	*Spirosoma*	49	Environmental	Bumble bee, Drone fly
	*Pseudomonas*	43	Gut/Environmental	Bumble bee, Drone fly, Honey bee
	*Snodgrassella*	40	Gut-associated	Bumble bee, Drone fly, Honey bee
	*Sphingomonas*	23	Environmental	Bumble bee, Drone fly
	*Dysgonomonas*	22	Environmental	Bumble bee, Drone fly, Honey bee
	*Pelomonas*	18	Environmental	Drone fly, Honey bee
	*Acinetobacter*	17	Gut/Environmental	Drone fly, Honey bee
	*Bombiscardovia*	16	Gut-associated	Bumble bee, Drone fly
	*Brevundimonas*	16	Environmental	Bumble bee, Drone fly, Honey bee
	*Enhydrobacter*	16	Environmental	Drone fly, Honey bee
2023	*Lactobacillus*	36	Gut-associated	Bumble bee, Native bee
	*Tepidimonas*	25	Environmental	Bumble bee, Native bee
	*Gilliamella*	23	Gut-associated	Bumble bee, Native bee
	*Snodgrassella*	19	Gut-associated	Bumble bee
	*Schlegelella*	16	Environmental	Bumble bee, Native bee
	*Hydrogenophilus*	14	Environmental	Bumble bee, Native bee
	*Ottowia*	14	Environmental	Bumble bee, Native bee
	*Pseudomonas*	12	Gut/Environmental	Bumble bee, Native bee
	*Meiothermus*	11	Environmental	Bumble bee, Native bee
	*Acinetobacter*	10	Gut/Environmental	Bumble bee, Native bee
	*Anaerobacillus*	10	Environmental	Bumble bee, Native bee
	*Sphingomonas*	10	Environmental	Bumble bee, Native bee

## Discussion

Distinct epibiont bacterial communities were observed across the four different pollinator species (bumble bees, honey bees, drone flies, and *L. sordidum*) visiting northern highbush blueberry plants, although some genera were shared between them. Interpretation of patterns involving drone flies should consider that, although they originated from wild‑collected pupae, adults were maintained under laboratory conditions before controlled blueberry exposure, whereas bees represent actively foraging or hive‑associated individuals; comparisons therefore reflect differences in life history and recent exposure context rather than direct ecological equivalence. In 2022, significant differences were observed in both the diversity and structure of the bacterial communities of the three pollinators (bumble bees, drone flies and honey bees) tested. Bumble bees had less diverse and compositionally distinct bacterial communities compared to drone flies and honey bees. Despite both bumble bees and honey bees being eusocial [32, 33], their beta diversity did not overlap, as seen in Fig. 1B NMDS, even though shared aspects of social organisation were expected to promote some similarity in epibiont bacterial communities. This lack of similarity suggests that differences in life history traits and behavioural ecology [4, 33, 34] play a stronger role in shaping epibiont bacterial acquisition, although replication of each pollinator system in different environments will be needed to generalise these findings. These interpretations assume that differences in pollinator behaviour (i.e. interaction with the flower) may influence external bacterial acquisition; this was not directly tested. Drone flies and honey bees exhibited similar levels of alpha diversity, yet their bacterial communities only partially overlapped. This overlap may be explained in part by the honey diet provided to laboratory‑reared drone adult flies while in captivity, potentially shifting their epibiont microbiota toward a honey bee–like composition, although such effects would be expected to primarily influence the gut microbiome rather than the epibiont community. Drone flies nonetheless exhibited the highest alpha diversity of the three species. This pattern is unlikely to be explained solely by their origin as wild‑collected pupae, as all pollinator species were exposed to environmental microbial sources before sampling, and persistence of larvae-acquired bacteria into adult epibiont communities was not assessed. A more plausible explanation is that drone fly epibiont communities are less host-associated and more strongly influenced by transient environmental acquisition, particularly given their laboratory maintenance before exposure. In 2023, similar patterns emerged: bumble bees again hosted a specialised and stable epibiont bacterial community compared to *L. sordidum*, reinforcing the tendency for bumble bees from the same hive to share similar bacterial profiles. In contrast, *L. sordidum*, being solitary rather than social bees, showed greater between-individual variability in epibiont bacterial composition. This likely reflects the broader environmental exposure, varied diets, and distinct ecological interactions of *L. sordidum* [35–37], which differ significantly from the hive-based lifestyles of bumble bees.

Overall, drone flies had the highest number of bacterial genera detected in their epibiont communities, whereas *L. sordidum* had the fewest, with bumble bees and honey bees being intermediate. Most bacterial genera detected on the epibiont were environmentally associated, likely originating from plants, water, or other environmental substrates previously encountered by the pollinators before the experimental setup and therefore persistent over time.

Nonetheless, several key pollinator gut-associated genera, *Lactobacillus*, *Gilliamella*, and *Snodgrassella* [3, 4, 7, 13] were detected in the epibiont of most pollinator species. Notably, this study reports the first detection of *Snodgrassella* on the drone fly *E. tenax*, and the first detection of *Gilliamella* and *Lactobacillus* on the native bee *L. sordidum*. Another gut-associated genus, *Bombiscardovia*, was also detected in bumble bee and drone fly samples of 2022, but at comparatively low relative abundance. The presence of these core gut-associated bacteria in the epibiont community suggests that some taxa may persist outside the gut environment [10] and may be transferred to host surfaces via faecal exposure, as demonstrated for *Snodgrassella* and *Gilliamella* in honey bees [38]. Although a previous study has suggested they could potentially contribute to similar ecological functions, such as pathogen resistance [10] these roles on the epibiont remain hypothetical and untested. In contrast, other well‑known pollinator gut‑associated genera, including *Bartonella*,* Schmidhempelia* and *Apibacter* [3] were not detected in the epibiont, implying that these taxa may be less capable of surviving or persisting outside the gut environment [10]. This absence is especially intriguing because *Apibacter* and *Chryseobacterium* are closely related [3, 39], yet only *Chryseobacterium* was detected in the samples. This pattern suggests that habitat conditions of the external surface strongly influence which bacterial genera are present on the epibiont of pollinators.

Finally, some genera, such as *Pseudomonas* and *Acinetobacter*, can be either gut-associated or environmentally derived, reflecting their ubiquity in nature [40, 41]. *Pseudomonas* inhabits a wide range of environments, including insect guts [6, 42] and external surfaces [10]. A*cinetobacter* includes species common in floral nectar (e.g., *A. nectaris*,* A. boissieri)*, while *A. apis* and *A. rathckeae* have been isolated from honey bee guts [43].

Flower‑ and plant‑associated bacteria are expected to form a major component of the environmentally acquired epibiont community. Although pollinators were observed visiting blueberry flowers within the cages, individual pollinator visitation could not be confirmed. As such, the detected epibiont communities reflect recent environmental exposure with likely, but not definitively verified, flower contact at the individual level. Since pollinators were not sterilised before introduction into the cages, previously acquired environmental bacteria likely contributed substantially to the observed epibiont communities. Pollinators likely acquire these bacteria through contact with floral nectaries and other flower structures during nectar foraging or pollen collection [16], creating opportunities for bacterial transmission. This is the likely origin of *Erwinia*,* Methylobacterium*, *Pantoea*, *Ralstonia*, and *Sphingomonas* [13, 44–46]. In eusocial pollinators, the hive also serves as a reservoir of epibiont bacteria, such as *Weissella*, which occurs in comb‑associated resources [47, 48].

Additional environmentally associated genera detected in this study include *Chryseobacterium*, linked to plants or soil [49]; and *Spirosoma*, associated with soil or plants [50]. Furthermore, *Hydrogenophilus*,* Meiothermus*, and *Tepidimonas* are thermophilic bacteria originating from high-temperature environments [51–53]. These genera were likely acquired transiently from the environment, most likely from soil or compost sludge.

The majority of the genera detected were not shared across sampling years, with only a subset of environmentally acquired genera present across both years. Because bumble bees were the only pollinator species sampled in both 2022 and 2023, interannual patterns primarily reflect observations from this species. Variation observed between sampling years in bumble bee epibiont communities is consistent with temporal variability in environmentally acquired bacteria, but does not distinguish between biological, environmental or methodological sources of variation. As sampling occurred during different flowering periods, this variation may instead reflect short‑term or phenology‑driven shifts in environmental bacterial exposure rather than true interannual turnover. Differences in exposure duration between years (6 h vs. 72 h) may have influenced the degree of bacterial accumulation on pollinator surfaces. Longer exposure likely increases contact with flowers and the surrounding environment, potentially contributing to variations observed between years. Temporal shifts in the gut microbiome have been documented in eusocial bees during the foraging season [54, 55]. Taken together, these observations are consistent with the possibility that the epibiont microbiome may, like the gut microbiome of eusocial bees, undergo seasonal shifts in bumble bees. Because sampling year and sequencing batch are confounded, and exposure duration differed between years, patterns observed across years should be introduced as descriptive rather than formal temporal comparisons.

As this study uses 16S rRNA gene sequencing, several inherent limitations should be acknowledged when interpreting the epibiont bacterial community. Key limitations of 16S rRNA sequencing include low taxonomic resolution (often to genus level) and a lack of information on organismal viability [13]. This limited the ability to identify species-specific bacteria, which would have given a clearer understanding of the potential environmental source of the bacteria. Since some genera can occur in multiple environments, subspecies-level identification is crucial for determining whether their activity is beneficial or pathogenic, as with *Pseudomonas*. Although a Basic Local Alignment Search Tool (BLAST) based approach can return high-identity species-level matches, conserved regions of the 16S rRNA V4 region often align to multiple closely related taxa, limiting taxonomic resolution [56]. Accordingly, species-level classification was not pursued, as the short read lengths generated in this study would not yield consistent or reliable assignments across the dataset. An approach that could instead improve would be to perform longer read sequencing using PacBio or Oxford Nanopore technologies, to identify species and maybe even subspecies [57]. Combining cell culture and plating, which provide information on bacterial viability, with long-read sequencing would allow for a deeper understanding of how environmental exposure shapes the epibiont microbiome of pollinators and should be explored in subsequent research. An additional limitation is the lack of multi-year replication for honey bees, drone flies, and *L. sordidum*, which restricts temporal interpretations for these species. Consequently, conclusions regarding seasonal or interannual dynamics are based solely on the bumble bee dataset.

Comparisons across pollinator species in this study are intended to highlight how epibiont bacterial communities vary across differing behavioural ecology systems (including social and foraging) and exposure histories linked to life history traits over a short-term common floral interaction, rather than to imply direct ecological equivalence among species. Together, these results highlight the substantial contribution of persistent environmentally acquired bacteria to pollinator epibiont communities. Future work should examine the environmental origins and persistence of these epibiont communities through geographically diverse sampling, environmental microbiome surveys, and temporal studies. Collectively, these findings illustrate how pollinator species identity, behavioural ecology, and life history traits shape the diversity and composition of epibiont bacterial communities across pollinator species, reflecting overlapping environmental exposure and acquisition potential, rather than direct evidence of plant–pollinator microbial exchange.

## Supplementary Information

Below is the link to the electronic supplementary material.

Supplementary Material 1 (DOCX 802 KB)

## Data Availability

The raw FASTQ files associated with this study are available through the NCBI Sequence Read Archive (SRA) under **PRJNA1498239**. Sequence data from the native bee *Lasioglossum sordidum* have been withheld from public release due to stewardship restrictions associated with Māori data sovereignty. The datasets generated and/or analysed during the current study are available in a GitHub repository and include the custom scripts and metatada: https://github.com/alignon/Pollinator-epibiont-bacterial-community.git.
