# Supplementary material for: Pollinator Species Identity, Behavioural Ecology and Life History Traits Shape Epibiont Bacterial Diversity
Source: Microb Ecol. 2026 Jul 23;89:191. doi: 10.1007/s00248-026-02845-9 (PMC13680588; doi:10.1007/s00248-026-02845-9)
Supplement: Supplementary file 1 [file 248_2026_2845_MOESM1_ESM.docx]

# Supplementary Information

**Pollinator species identity, behavioural ecology and life history traits shape epibiont bacterial diversity**

V. Aiko Lignon^1^, E. Eirian Jones^1^, Manpreet K. Dhami^1,2^, Clive Kaiser^3^ and Flore Mas^4^

^1^ Department of Pest-management and Conservation, Faculty of Agriculture and Life Sciences, Lincoln University, Lincoln, New Zealand

^2^ Bioeconomy Science Institute – Maiangi Taiao, PO Box 69040, Lincoln 7640, New Zealand

^3^ Department of Agricultural Sciences, Faculty of Agriculture and Life Sciences, Lincoln University, Lincoln, New Zealand

^4^ Bioeconomy Science Institute – Maiangi Taiao, Lincoln Science Centre, 74 Gerald Street, Lincoln 7608, New Zealand

Corresponding author: Aiko Lignon – aiko.lignon@lincolnuni.ac.nz

# Supplementary Figures

**Supplementary Fig. 1** Quality score profiles for some raw FASTQ reads showing per-cycle quality score distributions for forward and reverse reads A) in 2022 for bumble bee (*Bombus terrestris*), drone fly (*Eristalis tenax*) and honey bee (*Apis mellifera*) samples and B) in 2023 for bumble bee (B*. terrestris*) and native bee (*Lasioglossum sordidum*) samples**.** These profiles are used to assess read quality and guide truncation and filtering parameters in the DADA2 pipeline.

**Supplementary Fig 2.** Rarefaction curve representing the number of amplicon sequence variants (ASVs) and sequences for each sample in the pollinator samples. Each curve represents the enrichment in ASVs richness for a given sample size A) in 2022 for bumble bee (*Bombus terrestris*), drone fly (*Eristalis tenax*) and honey bee (*Apis mellifera*) samples and B) in 2023 for bumble bee (B*. terrestris*) and native bee (*Lasioglossum sordidum*) samples**.**

**Supplementary Fig. 3** Beta‑dispersion analysis of pollinator epibiont bacterial communities A) in 2022 for bumble bees (*Bombus terrestris*), drone flies (*Eristalis tenax*), and honey bees (*Apis mellifera*) (ANOVA, df = 2, *F* = 38.682, *p* = 2.81 × 10⁻¹¹) and B) in 2023 for bumble bees (*B. terrestris*) and native bees (*Lasioglossum sordidum*) (ANOVA, df = 1, *F* = 12.808, *p* = 0.00145). Principal Coordinates Analysis (PCoA) based on Bray–Curtis dissimilarities among pollinator species, with lines connecting each sample to its group centroid. Boxplots show distances of samples to their respective centroids, illustrating multivariate dispersion.

# Supplementary Tables

**Supplementary Table 1:** Summary of sequencing read counts at each processing step (input, filtering, denoising of forward and reverse reads, merging, and chimera removal) for all samples, including controls. Controls comprise a DNA extraction negative control (**DNAnegative** = MilliQ water), a PCR mock community positive control (**Mock_positive**), and a PCR no‑template negative control (**Negative** = MilliQ water).

| Year | Sample name | Input | Filtered | DenoisedF | DenoisedR | Merged | Nonchim |
| --- | --- | --- | --- | --- | --- | --- | --- |
| 2022 | 2022Pollinator1a | 972 | 952 | 943 | 945 | 941 | 941 |
|  | 2022Pollinator1b | 510 | 491 | 476 | 473 | 467 | 467 |
|  | 2022Pollinator1c | 1124 | 1068 | 1060 | 1058 | 1058 | 1058 |
|  | 2022Pollinator1d | 2143 | 2049 | 2028 | 2024 | 2024 | 2024 |
|  | 2022Pollinator2a | 75473 | 73703 | 73570 | 73479 | 73258 | 72364 |
|  | 2022Pollinator2b | 2350 | 2292 | 2284 | 2279 | 2265 | 2265 |
|  | 2022Pollinator2c | 44716 | 42837 | 42757 | 42743 | 42441 | 42304 |
|  | 2022Pollinator2d | 11543 | 11325 | 11311 | 11303 | 11244 | 11179 |
|  | 2022Pollinator3a | 25 | 24 | 18 | 21 | 18 | 18 |
|  | 2022Pollinator3b | 9 | 9 | 6 | 6 | 6 | 6 |
|  | 2022Pollinator3c | 242 | 238 | 232 | 229 | 229 | 229 |
|  | 2022Pollinator3d | 42147 | 41464 | 41402 | 41362 | 41285 | 40496 |
|  | 2022Pollinator4a | 13882 | 13553 | 13526 | 13519 | 13409 | 13312 |
|  | 2022Pollinator4b | 3457 | 3396 | 3385 | 3378 | 3283 | 3283 |
|  | 2022Pollinator4c | 176 | 159 | 141 | 144 | 133 | 133 |
|  | 2022Pollinator4d | 1729 | 1696 | 1683 | 1672 | 1649 | 1649 |
|  | 2022Pollinator5b | 23 | 21 | 15 | 15 | 15 | 15 |
|  | 2022Pollinator5c | 1 | 1 | 1 | 1 | 1 | 1 |
|  | 2022Pollinator5d | 654 | 629 | 609 | 600 | 581 | 581 |
|  | 2022Pollinator6a | 15729 | 15367 | 15294 | 15278 | 15000 | 14182 |
|  | 2022Pollinator6b | 819 | 796 | 779 | 773 | 772 | 772 |
|  | 2022Pollinator6c | 56177 | 54056 | 53901 | 53843 | 53446 | 52655 |
|  | 2022Pollinator6d | 15994 | 15680 | 15661 | 15633 | 15512 | 15254 |
|  | 2022Pollinator7a | 2293 | 2227 | 2217 | 2218 | 2198 | 2196 |
|  | 2022Pollinator7b | 565 | 530 | 513 | 513 | 509 | 509 |
|  | 2022Pollinator7c | 11 | 11 | 6 | 6 | 6 | 6 |
|  | 2022Pollinator7d | 3303 | 3243 | 3241 | 3226 | 3205 | 3203 |
|  | 2022Pollinator8a | 12758 | 12087 | 12081 | 12075 | 12057 | 12057 |
|  | 2022Pollinator8b | 895 | 878 | 876 | 873 | 873 | 873 |
|  | 2022Pollinator8c | 118437 | 116034 | 115864 | 115816 | 114837 | 111410 |
|  | 2022Pollinator8d | 507 | 496 | 480 | 491 | 473 | 471 |
|  | 2022Pollinator9a | 8 | 7 | 6 | 6 | 6 | 6 |
|  | 2022Pollinator9b | 402 | 390 | 385 | 382 | 382 | 382 |
|  | 2022Pollinator10b | 8109 | 7734 | 7709 | 7717 | 7592 | 7592 |
|  | 2022Pollinator10c | 2497 | 2440 | 2432 | 2432 | 2430 | 2430 |
|  | 2022Pollinator10d | 1567 | 1526 | 1524 | 1524 | 1524 | 1524 |
|  | 2022Pollinator11a | 311 | 289 | 284 | 284 | 284 | 284 |
|  | 2022Pollinator11b | 29 | 26 | 20 | 20 | 20 | 20 |
|  | 2022Pollinator12a | 24 | 24 | 23 | 19 | 13 | 13 |
|  | 2022Pollinator12b | 1172 | 1070 | 1070 | 1069 | 1069 | 1069 |
|  | 2022Pollinator12c | 15461 | 15106 | 15100 | 15094 | 15091 | 15091 |
|  | 2022Pollinator12d | 490 | 460 | 453 | 452 | 452 | 452 |
|  | 2022Pollinator13a | 697062 | 629401 | 628078 | 626569 | 618740 | 598300 |
|  | 2022Pollinator13b | 6223 | 6044 | 6036 | 6009 | 5927 | 5898 |
|  | 2022Pollinator13c | 403 | 370 | 353 | 349 | 323 | 323 |
|  | 2022Pollinator13d | 8 | 7 | 5 | 4 | 4 | 4 |
|  | 2022Pollinator14a | 2745 | 2653 | 2650 | 2648 | 2648 | 2648 |
|  | 2022Pollinator14b | 21097 | 20287 | 20222 | 20249 | 20189 | 20101 |
|  | 2022Pollinator14c | 86550 | 83634 | 83594 | 83529 | 83371 | 83360 |
|  | 2022Pollinator14d | 8605 | 8340 | 8312 | 8309 | 8302 | 8191 |
|  | 2022Pollinator15a | 92984 | 87360 | 87152 | 86814 | 85736 | 83461 |
|  | 2022Pollinator15b | 1601 | 1521 | 1516 | 1476 | 1450 | 1442 |
|  | 2022Pollinator15c | 6 | 6 | 2 | 2 | 2 | 2 |
|  | 2022Pollinator15d | 20 | 19 | 14 | 13 | 13 | 13 |
|  | 2022Pollinator16a | 4601 | 4395 | 4390 | 4389 | 4389 | 4386 |
|  | 2022Pollinator16b | 2812 | 2713 | 2710 | 2710 | 2710 | 2710 |
|  | 2022Pollinator16c | 12427 | 11987 | 11966 | 11965 | 11933 | 11933 |
|  | 2022Pollinator16d | 14434 | 11596 | 11515 | 11537 | 11463 | 11463 |
|  | 2022Pollinator17a | 45522 | 41332 | 41249 | 41274 | 41054 | 39688 |
|  | 2022Pollinator17b | 3443 | 3293 | 3246 | 3279 | 3185 | 3134 |
|  | 2022Pollinator17c | 3847 | 3674 | 3651 | 3638 | 3585 | 3580 |
|  | 2022Pollinator17d | 176 | 137 | 131 | 134 | 130 | 130 |
|  | 2022Pollinator18a | 2519 | 2415 | 2414 | 2414 | 2403 | 2403 |
|  | 2022Pollinator18b | 209029 | 196134 | 195949 | 195678 | 194195 | 193159 |
|  | 2022Pollinator18c | 44396 | 42986 | 42971 | 42947 | 42891 | 42891 |
|  | 2022Pollinator19a | 19942 | 19170 | 19147 | 19132 | 19073 | 19073 |
|  | 2022Pollinator19b | 110329 | 100703 | 100590 | 100556 | 100271 | 99959 |
|  | 2022Pollinator19c | 5499 | 5361 | 5337 | 5336 | 5335 | 5335 |
|  | 2022Pollinator19d | 1 | 1 | 1 | 1 | 1 | 1 |
|  | 2022Pollinator20a | 1390 | 1320 | 1319 | 1319 | 1318 | 1291 |
|  | 2022Pollinator20b | 18089 | 17263 | 17243 | 17244 | 17227 | 17227 |
|  | 2022DNAnegative1 | 48586 | 46664 | 46619 | 46574 | 46480 | 45453 |
|  | 2022DNAnegative2 | 3 | 3 | 2 | 1 | 1 | 1 |
|  | 2022DNAnegative3 | 40441 | 37860 | 37808 | 37758 | 37534 | 37534 |
|  | 2022DNAnegative4 | 4547 | 4421 | 4384 | 4415 | 4374 | 4374 |
|  | 2022DNAnegative5 | 14676 | 13906 | 13900 | 13894 | 13830 | 13830 |
|  | 2022DNAnegative6 | 13138 | 12795 | 12786 | 12779 | 12776 | 12776 |
|  | 2022DNAnegative7 | 7209 | 6944 | 6940 | 6849 | 6793 | 6790 |
|  | 2022DNAnegative8 | 2511 | 2449 | 2448 | 2446 | 2446 | 2401 |
|  | 2022DNAnegative9 | 32907 | 30327 | 30301 | 30266 | 30179 | 29051 |
|  | 2022DNAnegative10 | 4332 | 4206 | 4203 | 4194 | 4193 | 4193 |
|  | 2022DNAnegative11 | 5978 | 5854 | 5842 | 5841 | 5838 | 5838 |
|  | 2022DNAnegative12 | 2385 | 2333 | 2325 | 2319 | 2318 | 2318 |
|  | 2022Mock_Positive1 | 1087487 | 995005 | 992733 | 992265 | 916293 | 624117 |
|  | 2022Mock_Positive2 | 95771 | 89393 | 88873 | 88908 | 79658 | 50320 |
|  | 2022Mock_Positive3 | 100943 | 93654 | 93286 | 93230 | 87072 | 68086 |
|  | 2022Mock_Positive4 | 302699 | 279132 | 278639 | 278466 | 258570 | 190400 |
|  | 2022Mock_Positive5 | 109 | 102 | 98 | 98 | 96 | 96 |
|  | 2022Negative2 | 1 | 1 | 1 | 1 | 0 | 0 |
|  | 2022Negative6 | 3 | 3 | 3 | 3 | 3 | 3 |
| 2023 | 2023Pollinator1a | 1876 | 1804 | 1804 | 1803 | 1789 | 1789 |
|  | 2023Pollinator1b | 4262 | 4142 | 4134 | 4121 | 4107 | 4103 |
|  | 2023Pollinator1c | 62 | 56 | 48 | 48 | 48 | 48 |
|  | 2023Pollinator1d | 16405 | 15935 | 15867 | 15848 | 15734 | 15685 |
|  | 2023Pollinator2a | 5232 | 5112 | 5096 | 5074 | 5039 | 5036 |
|  | 2023Pollinator2b | 864 | 841 | 841 | 841 | 841 | 841 |
|  | 2023Pollinator2c | 11206 | 10657 | 10648 | 10596 | 10483 | 10454 |
|  | 2023Pollinator2d | 643 | 637 | 616 | 623 | 604 | 604 |
|  | 2023Pollinator3a | 100 | 98 | 95 | 94 | 94 | 94 |
|  | 2023Pollinator3b | 146 | 142 | 137 | 134 | 127 | 116 |
|  | 2023Pollinator3c | 881 | 844 | 831 | 825 | 820 | 820 |
|  | 2023Pollinator3d | 10008 | 9230 | 9216 | 9198 | 9140 | 9138 |
|  | 2023Pollinator4a | 5674 | 5495 | 5492 | 5488 | 5486 | 5486 |
|  | 2023Pollinator4b | 3387 | 3215 | 3208 | 3208 | 3181 | 3061 |
|  | 2023Pollinator4c | 769 | 724 | 720 | 717 | 699 | 699 |
|  | 2023Pollinator4d | 1002 | 978 | 975 | 941 | 935 | 935 |
|  | 2023Pollinator5a | 71 | 68 | 61 | 53 | 48 | 48 |
|  | 2023Pollinator5b | 13 | 12 | 7 | 7 | 7 | 7 |
|  | 2023Pollinator5c | 23 | 22 | 19 | 19 | 19 | 19 |
|  | 2023Pollinator5d | 8563 | 8015 | 7982 | 7883 | 7788 | 7777 |
|  | 2023Pollinator6a | 129378 | 126550 | 126247 | 126132 | 124786 | 120930 |
|  | 2023Pollinator6b | 36840 | 36059 | 35981 | 35868 | 35617 | 35103 |
|  | 2023Pollinator6c | 761821 | 725215 | 724369 | 723282 | 711220 | 687069 |
|  | 2023Pollinator6d | 220789 | 212710 | 212389 | 212154 | 210734 | 208757 |
|  | 2023Pollinator7a | 77754 | 72996 | 72971 | 72938 | 72915 | 72875 |
|  | 2023Pollinator7b | 510 | 472 | 471 | 471 | 471 | 471 |
|  | 2023Pollinator8a | 5410 | 5256 | 5251 | 5253 | 5251 | 5251 |
|  | 2023Pollinator9a | 28205 | 27175 | 27144 | 27116 | 26861 | 25006 |
|  | 2023Pollinator9b | 25099 | 23746 | 23717 | 23666 | 23563 | 23492 |
|  | 2023Pollinator10a | 39346 | 38049 | 38022 | 37914 | 37701 | 37035 |
|  | 2023Pollinator11a | 172385 | 166518 | 166406 | 166029 | 165391 | 164891 |
|  | 2023Pollinator12a | 12528 | 10235 | 10226 | 10223 | 10221 | 10221 |
|  | 2023Pollinator12b | 4941 | 4690 | 4681 | 4675 | 4555 | 4547 |
|  | 2023DNAnegative2 | 117 | 113 | 110 | 110 | 110 | 110 |
|  | 2023DNAnegative4 | 53729 | 51498 | 51465 | 51473 | 51352 | 50128 |
|  | 2023DNAnegative5 | 6 | 6 | 3 | 3 | 3 | 3 |
|  | 2023DNAnegative6 | 343 | 333 | 330 | 330 | 330 | 330 |
|  | 2023Mock_Positive1 | 47261 | 39212 | 38993 | 39045 | 36540 | 30364 |
|  | 2023Mock_Positive2 | 58086 | 54730 | 54165 | 54493 | 52432 | 40713 |
|  | 2023Mock_Positive3 | 7076 | 6600 | 6490 | 6502 | 6023 | 5093 |
|  | 2023Mock_Positive4 | 52990 | 49237 | 49011 | 48954 | 46428 | 37524 |
|  | 2023Negative1 | 23201 | 21692 | 21681 | 21372 | 21324 | 21307 |
|  | 2023Negative2 | 13015 | 12532 | 12496 | 12526 | 12486 | 12486 |
|  | 2023Negative3 | 33 | 14 | 11 | 11 | 11 | 11 |
|  | 2023Negative4 | 10367 | 10108 | 10106 | 10101 | 10098 | 10055 |
|  | 2023Negative5 | 33 | 31 | 26 | 28 | 26 | 26 |

**Supplementary Table 2**: Previously identified bacteria species found associated (gut microbiome or surface) with the pollinators used in this study from the literature and their reference.

| Sample Source | Bacteria Species | Reference |
| --- | --- | --- |
| Bee (*Apis* and *Bombus* ssp.) – gut | *Gilliamella apicola*  *Snodgrassella alvi*  *Lactobacillus* sp.  *Bifidobacterium* sp. | Moran (2015) |
| Bee (*Apis* sp.) - gut | *Caulobacter*  *Escherichia*  *Gilliamella*  *Snodgrasella*  *Lactobacillus* | Graystock et al. (2017) |
| Bee – pathogens | *Paenibacillus larvae (AFB)*  *Melissococcus plutonius (EFB)*  *Serratia marcescens*  *Lysinibacillus sphaericus* | Fünfhaus et al. (2018) |
| Honey bee (*Apis mellifera*) - gut | *Gilliamella apicola,*  *Frischella perrara,*  *Snodgrassella alvi,*  [*Bartonella*](https://www.sciencedirect.com/topics/agricultural-and-biological-sciences/bartonella) *apis*  [*Lactobacillus*](https://www.sciencedirect.com/topics/agricultural-and-biological-sciences/lactobacillus) spp.  [*Bifidobacterium*](https://www.sciencedirect.com/topics/agricultural-and-biological-sciences/bifidobacterium)  *Parasaccharibacter apium* | Miller et al. (2019) |
| Bumble bee (*Bombus terrestris*) – gut | *Acinetobacter* sp.  *Bombiscardovia* sp  *Chryseobacterium* sp.  *Escherichia coli*  *Fructobacillus* sp.  *Gluconobacter* sp.  *Lactobacillus* sp.  *Pseudomonas* sp.  *Pseudomonas fragi*  *Snodgrassella alvi* | Felden et al. (2021) |
| Halictine bee (*Lasioglossum* sp.) – gut and surface | *Lactobacillaceae*  *Enterobacteriaceae (Sodalis)*  *Rickettsiaceae*  *Comamonadeaceae,*  *Moraxellaceae,*  *Oxalobacteraceae,*  *Pseudomonaceae*  *Xanthomonadaceae*  *Alcaligenaceae* | Mayr et al. (2021) |
| Honey bee (*Apis mellifera*) – whole bee | *Lactobacillus* spp.  *Arsenophonus* sp.  *Gilliamella* spp.  *Bifidobacterium* spp.  *Snodgrassella* spp.  *Bartonella* spp.  *Frischella* spp.  *Commensalibacter* sp.  *Bombilactobacillus* sp.  *Pseudomonas* sp.  *Apibacter* sp.  *Rhizobiaceae* sp. | Almeida et al. (2023) |
| Honey bee (*Apis mellifera*) – gut | *Lactobacillus* spp.  *Bifidobacterium asteroids*  *Snodgrasella alvi*  *Gilliamella apicola*  *Frischella perrara*  *Bombella apis* | Anderson et al. (2023) |
| Bumble bee (*Bombus terrestris*) – gut | *Gilliamella* spp.  *Apibacter mensalis*  *Lactobacillus* spp.  *Bombiscardovia* sp.  *Bradyrhizobium* sp.  *Bifidobacterium* spp.  *Caulobacter* sp.  *Sphingomonas* sp.  *Burkholderia-Caballeronia-Paraburkholderia* sp.  *Staphylococcus carnosus*  *Halomonas* sp.  *Enhydrobacter* sp. | Choi et al. (2023) |
| Wild bee (*Lasioglossum*) – gut | *Pseudomonas*  *Sodalis*  *Lactobacillus*  *Wolbachia*  *Snodgrassella*  *Rhizobium*  *Enterobacter*  *Gilliamella*  *Sphingobium* | Fernandez De Landa et al. (2023) |
| Bumble bee (*Bombus impatiens*) – gut | *Lactobacillus*  *Apilactobacillus*  *Schmidhempelia*  *Gilliamella*  *Snodgrassella*  *Bombilactobacillus*  *Bombiscardovia* | Hammer et al. (2023) |
| Bumble bee (*Bombus* spp.), honey bee (*Apis* sp.) and fly (*Eristalis* sp.) – gut | *Gilliamella* sp.  *Snodgrassella* sp.  *Lactobacillus* sp. | Li et al. (2023) |
| Honey bee *(Apis* spp.) and red mason bee (*Osmia bicornis*) – cuticle | *Bacteroides* sp.  *Bifidobacterium* sp.  *Citrobacter* sp.  *Clostridium sensu stricto 1* sp.  *Gilliamella* sp.  *Lactobacillus* sp.  *Melissococcus* sp.  *Microbacterium* sp.  *Microlunatus* sp.  *Pelomonas* sp.  *Pseudomonas* sp.  *Ruminococcus* sp.  *Saccharibacter* sp.  *Snodgrassella* sp.  *Spiroplasma* sp.  And other species | Thamm et al. (2023) |
| Wild bumble bee (*Bombus pauloensis* and *B. bellicosus*) - gut | *Pseudomonas*  *Snodrgrasella*  *Gilliamella*  *Lactobacillus*  *Wolbachia*  *Sodalis*  *Bifidobacterium*  *Spiroplasma*  *Apibacter*  *Pantoea*  *Rosenbergiella*  *Arsenophonus* | Fernandez De Landa et al. (2024) |
| Honey bee (*Apis* spp.) and bumble bee (*Bombus* sp.) – gut | *Snodgrassella* sp.  *Gilliamella* sp.  *Bartonella* sp.  *Schmidhempelia* sp.  *Frischella* sp.  *Lactobacillus* sp.  *Bifidobacterium* sp.  *Apibacter* sp. | Yang et al. (2025) |

The reference list used in genus analysis was compiled based on how often a genus taxon was present in the table. *Wolbachia* and *Sodalis* are insect symbionts that cannot survive outside of their host (Bourtzis & Miller, 2003), so they were excluded from the reference list. Additionally, if a genus is well documented to be plant-associated, like *Rosenbergiella* (Vannette, 2020), it was removed from the reference list.

**Supplementary Table 3:** Previously identified bacteria species found in blueberry flowers or nectar from published data and the reference. Genera shown in bold occur in both Supplementary Tables 1 and 2 and were therefore considered as either gut‑associated or environmentally acquired.

| Sample source | Bacteria Species | Reference |
| --- | --- | --- |
| Blueberry (*Vaccinium corymbosum* 'Snowchaser') flower | - *Rosenbergiella epipactidis* - ***Pseudomonas*** *tremae* - *Microbacterium testaceum* - *Xylophilus ampelinus* - *Curtobacterium pusillum* - *Fictibacillus phosphorivorans* - ***Serratia*** *marcescens* - *Asaia spathodeae* | Chacón et al. (2022) |
| Blueberry (*Vaccinium corymbosum ‘*Meadowlark’ and ‘Arcadia’) and shiny blueberry (*Vaccinium myrsinites)* nectar | - *Neokomagataea thailandica* - *Rosenbergiella* sp. - *Pantoea agglomerans* - *Bacillus* sp. - *Kocuria rhizophila* - ***Acinetobacter*** *apis* - ***Acinetobacter*** *nectaris* - *Erwinia aphidicola* - *Neokomagataea tanensis* - *Acinetobacter boissieri* - *Lysinibacillus chungkukjangi* - ***Acinetobacter*** *radioresistens* - ***Pseudomonas*** sp. - *Bacillus nealsonii* - *Gluconobacter wancherniae* | Rering et al. (2024) |

**Supplementary Table 4**: Results of a permutational multivariate analysis of variance (PERMANOVA) using Bray–Curtis dissimilarities to assess differences in epibiont bacterial community composition across the different pollinator species in 2022 for bumble bee (*Bombus terrestris*), drone fly (*Eristalis tenax*), honey bee (*Apis mellifera*) and in 2023 for bumble bee (*B. terrestris*) and native bee (*Lasioglossum sordidum*). The analysis was based on 999 permutations. Significant p-values (p < 0.001) are shown in bold.

| Year |  | Df | Sum of Squares | R^2^ | *F*-value | *p*-value |
| --- | --- | --- | --- | --- | --- | --- |
| 2022 | Model | 2 | 5.4054 | 0.25896 | 9.7847 | **0.001** |
|  | Residual | 56 | 15.4682 | 0.74104 |  |  |
| 2023 | Model | 1 | 1.8373 | 0.21949 | 7.0304 | **0.001** |
|  | Residual | 25 | 6.5333 | 0.7851 |  |  |

**Supplementary Table 5**: Most frequently detected genera by amplicon sequence variants (ASVs) (n ≤ 5) in the epibiont bacterial communities of each pollinator species in 2022 for bumble bees (*Bombus terrestris*), drone flies (*Eristalis tenax*), and honey bees (*Apis mellifera*). Each genus is classified as gut‑associated, environmental, or gut/environmental to indicate the potential source from which it was acquired and the pollinator species in which it was detected. Bold genus = Genus found in 2022 and 2023 (Supplementary Tables 3 and 4).

| **Genus** | **ASV Frequency in Samples** | **Classification** | **Pollinator Species Found in** |
| --- | --- | --- | --- |
| ***Lactobacillus*** | 60 | Gut-associated | Bumble bee, Drone fly, Honey bee |
| ***Gilliamella*** | 50 | Gut-associated | Bumble bee, Drone fly, Honey bee |
| *Spirosoma* | 49 | Environmental | Bumble bee, Drone fly |
| ***Pseudomonas*** | 43 | Gut/ Environmental | Bumble bee, Drone fly, Honey bee |
| ***Snodgrassella*** | 40 | Gut-associated | Bumble bee, Drone fly, Honey bee |
| ***Sphingomonas*** | 23 | Environmental | Bumble bee, Drone fly |
| *Dysgonomonas* | 22 | Environmental | Bumble bee, Drone fly, Honey bee |
| *Pelomonas* | 18 | Environmental | Drone fly, Honey bee |
| ***Acinetobacter*** | 17 | Gut/ Environmental | Drone fly, Honey bee |
| *Bombiscardovia* | 16 | Gut-associated | Bumble bee, Drone fly |
| *Brevundimonas* | 16 | Environmental | Bumble bee, Drone fly, Honey bee |
| *Enhydrobacter* | 16 | Environmental | Drone fly, Honey bee |
| ***Klebsiella*** | 14 | Environmental | Drone fly, Honey bee |
| *Serratia* | 13 | Gut/ Environmental | Bumble bee, Drone fly |
| *Vagococcus* | 13 | Environmental | Bumble bee, Drone fly, Honey bee |
| ***Massilia*** | 10 | Environmental | Drone fly |
| ***Methylobacterium*** | 10 | Environmental | Drone fly, Honey bee |
| *Xylophilus* | 10 | Environmental | Bumble bee, Drone fly, Honey bee |
| *Burkholderia-Caballeronia-Paraburkholderia* | 9 | Environmental | Drone fly, Honey bee |
| *Escherichia/Shigella* | 9 | Environmental | Drone fly |
| *Ralstonia* | 9 | Environmental | Drone fly, Honey bee |
| *Zymobacter* | 9 | Environmental | Bumble bee, Drone fly |
| *Hymenobacter* | 8 | Environmental | Honey bee |
| ***Weissella*** | 8 | Environmental | Bumble bee |
| *Hafnia-Obesumbacterium* | 7 | Environmental | Drone fly, Honey bee |
| *Lawsonella* | 7 | Environmental | Drone fly |
| *Micrococcus* | 7 | Environmental | Drone fly |
| *Proteus* | 7 | Environmental | Drone fly, Honey bee |
| ***Anaerobacillus*** | 6 | Environmental | Drone fly, Honey bee |
| ***Bradyrhizobium*** | 6 | Environmental | Drone fly, Honey bee |
| ***Chryseobacterium*** | 6 | Environmental | Bumble bee, Drone fly |
| ***Pantoea*** | 6 | Environmental | Drone fly, Honey bee |
| *Sphingopyxis* | 6 | Environmental | Drone fly, Honey bee |
| *Atopostipes* | 5 | Environmental | Honey bee |
| *Ellin6055* | 5 | Environmental | Drone fly, Honey bee |
| *Enterococcus* | 5 | Environmental | Drone fly |
| *Fusobacterium* | 5 | Environmental | Drone fly |
| *Galbitalea* | 5 | Environmental | Drone fly |
| *Hyphomicrobium* | 5 | Environmental | Honey bee |
| *Morganella* | 5 | Environmental | Drone fly |

**Supplementary Table 6**: Most frequently detected genera by amplicon sequence variants (ASVs) (n ≤ 5) in the epibiont bacterial communities of each pollinator species in 2023 for bumble bees (*Bombus terrestris*) and native bees (*Lasioglossum sordidum*). Each genus is classified as gut‑associated, environmental, or gut/environmental to indicate the potential source from which it was acquired and the pollinator species in which it was detected. Bold genus = Genus found in 2022 and 2023 (Supplementary Tables 3 and 4).

| **Genus** | **ASV Frequency in Samples** | **Classification** | **Pollinator Species Found in** |
| --- | --- | --- | --- |
| ***Lactobacillus*** | 36 | Gut-associated | Bumble bee, Native bee |
| *Tepidimonas* | 25 | Environmental | Bumble bee, Native bee |
| ***Gilliamella*** | 23 | Gut-associated | Bumble bee, Native bee |
| ***Snodgrassella*** | 19 | Gut-associated | Bumble bee |
| *Schlegelella* | 16 | Environmental | Bumble bee, Native bee |
| *Hydrogenophilus* | 14 | Environmental | Bumble bee, Native bee |
| *Ottowia* | 14 | Environmental | Bumble bee, Native bee |
| ***Pseudomonas*** | 12 | Gut/ Environmental | Bumble bee, Native bee |
| *Meiothermus* | 11 | Environmental | Bumble bee, Native bee |
| ***Acinetobacter*** | 10 | Gut/ Environmental | Bumble bee, Native bee |
| ***Anaerobacillus*** | 10 | Environmental | Bumble bee, Native bee |
| ***Sphingomonas*** | 10 | Environmental | Bumble bee, Native bee |
| ***Chryseobacterium*** | 9 | Environmental | Bumble bee, Native bee |
| *Corynebacterium_1* | 7 | Environmental | Bumble bee, Native bee |
| ***Massilia*** | 6 | Environmental | Bumble bee, Native bee |
| ***Pantoea*** | 6 | Environmental | Bumble bee |
| *Sphingobium* | 6 | Environmental | Bumble bee, Native bee |
| ***Bradyrhizobium*** | 5 | Environmental | Bumble bee, Native bee |
| *Erwinia* | 5 | Environmental | Bumble bee |
| ***Klebsiella*** | 5 | Environmental | Bumble bee |
| ***Methylobacterium*** | 5 | Environmental | Bumble bee, Native bee |
| *Nocardioides* | 5 | Environmental | Bumble bee, Native bee |
| *Vulcaniibacterium* | 5 | Environmental | Bumble bee, Native bee |
| ***Weissella*** | 5 | Environmental | Bumble bee |

# References

Almeida, E. L., Ribiere, C., Frei, W., Kenny, D., Coffey, M. F., & O’Toole, P. W. (2023). Geographical and Seasonal Analysis of the Honeybee Microbiome. *Microbial Ecology, 85*(2), 765-778. <https://doi.org/10.1007/s00248-022-01986-x>

Anderson, K. E., Ricigliano, V. A., Copeland, D. C., Mott, B. M., & Maes, P. (2023). Social Interaction is Unnecessary For Hindgut Microbiome Transmission in Honey Bees: The Effect of Diet and Social Exposure on Tissue-Specific Microbiome Assembly. *Microbial Ecology, 85*(4), 1498-1513. <https://doi.org/10.1007/s00248-022-02025-5>

Bourtzis, K., & Miller, T. A. (2003). *Insect Symbiosis* (Vol. 1). CRC Press.

Chacón, F. I., Sineli, P. E., Mansilla, F. I., Pereyra, M. M., Diaz, M. A., Volentini, S. I., Poehlein, A., Meinhardt, F., Daniel, R., & Dib, J. R. (2022). Native cultivable bacteria from the blueberry microbiome as novel potential biocontrol agents. *Microorganisms, 10*(5), 969. <https://www.mdpi.com/2076-2607/10/5/969>

Choi, H., Roy, N., Kim, J.-M., Joo Yoon, H., Yong Lee, K., Lee, K.-S., & Choi, K. (2023). Dynamics of Gut Microbiome Upon Pollination in Bumblebee (*Bombus terrestris*). *Journal of Asia-Pacific Entomology, 26*(1), 102042. <https://doi.org/10.1016/j.aspen.2023.102042>

Felden, A., Baty, J. W., & Lester, P. J. (2021). Gut microbial communities and pathogens infection in New Zealand bumble bees (*Bombus terrestris*, Linnaeus, 1758). *New Zealand Entomologist, 44*(2), 71-80. <https://doi.org/10.1080/00779962.2022.2053350>

Fernandez De Landa, G., Alberoni, D., Baffoni, L., Fernandez De Landa, M., Revainera, P. D., Porrini, L. P., Brasesco, C., Quintana, S., Zumpano, F., Eguaras, M. J., Maggi, M. D., & Di Gioia, D. (2023). The gut microbiome of solitary bees is mainly affected by pathogen assemblage and partially by land use. *Environmental Microbiome, 18*(1), 38. <https://doi.org/10.1186/s40793-023-00494-w>

Fernandez De Landa, G., Alberoni, D., Braglia, C., Baffoni, L., Fernandez de Landa, M., Revainera, P. D., Quintana, S., Zumpano, F., Maggi, M. D., & Di Gioia, D. (2024). The Gut Microbiome of Two Wild Bumble Bee Species Native of South America: *Bombus pauloensis* and *Bombus bellicosus*. *Microbial Ecology, 87*(1), 121. <https://doi.org/10.1007/s00248-024-02430-y>

Fünfhaus, A., Ebeling, J., & Genersch, E. (2018). Bacterial Pathogens of Bees. *Current Opinion in Insect Science, 26*, 89-96. <https://doi.org/https://doi.org/10.1016/j.cois.2018.02.008>

Graystock, P., Rehan, S. M., & McFrederick, Q. S. (2017). Hunting For Healthy Microbiomes: Determining the Core Microbiomes of *Ceratina, Megalopta*, and *Apis* Bees and How they Associate With Microbes in Bee Collected Pollen. *Conservation Genetics, 18*(3), 701-711. <https://doi.org/10.1007/s10592-017-0937-7>

Hammer, T. J., Easton-Calabria, A., & Moran, N. A. (2023). Microbiome Assembly and Maintenance Across the Lifespan of Bumble Bee Workers. *Molecular Ecology, 32*(3), 724-740. <https://doi.org/https://doi.org/10.1111/mec.16769>

Li, J., Sauers, L., Zhuang, D., Ren, H., Guo, J., Wang, L., Zhuang, M., Guo, Y., Zhang, Z., Wu, J., Yao, J., Yang, H., Huang, J., Wang, C., Lin, Q., Zhang, Z., & Sadd, B. M. (2023). Divergence and convergence of gut microbiomes of wild insect pollinators. *mBio, 14*(4), e01270-01223. <https://doi.org/doi:10.1128/mbio.01270-23>

Mayr, A. V., Keller, A., Peters, M. K., Grimmer, G., Krischke, B., Geyer, M., Schmitt, T., & Steffan-Dewenter, I. (2021). Cryptic Species and Hidden Ecological Interactions of Halictine Bees Along an Elevational Gradient. *Ecology and Evolution, 11*(12), 7700-7712. <https://doi.org/https://doi.org/10.1002/ece3.7605>

Miller, D. L., Parish, A. J., & Newton, I. L. G. (2019). Transitions and Transmission: Behaviour and Physiology as Drivers of Honey Bee-Associated Microbial Communities. *Current opinion in microbiology, 50*, 1-7. <https://doi.org/https://doi.org/10.1016/j.mib.2019.08.001>

Moran, N. A. (2015). Genomics of the Honey Bee Microbiome. *Current Opinion in Insect Science, 10*, 22-28. <https://doi.org/https://doi.org/10.1016/j.cois.2015.04.003>

Rering, C. C., Rudolph, A. B., Li, Q.-B., Read, Q. D., Muñoz, P. R., Ternest, J. J., & Hunter, C. T. (2024). A quantitative survey of the blueberry (*Vaccinium* spp.) culturable nectar microbiome: Variation between cultivars, locations, and farm management approaches. *FEMS Microbiology Ecology, 100*(3). <https://doi.org/10.1093/femsec/fiae020>

Thamm, M., Reiß, F., Sohl, L., Gabel, M., Noll, M., & Scheiner, R. (2023). Solitary bees host more bacteria and fungi on their cuticle than social bees. *Microorganisms, 11*(11). <https://doi.org/10.3390/microorganisms11112780>

Vannette, R. L. (2020). The floral microbiome: Plant, pollinator, and microbial perspectives. *Annual Review of Ecology, Evolution, and Systematics, 51*, 363-386.

Yang, C., Hu, J., Su, Q., Zhang, Z., Du, Y., Wang, J., Sun, H., Han, B., Tang, J., Guo, L., Li, H., Cai, W., Zheng, H., Zhou, X., & Zhang, X. (2025). A review on recent taxonomic updates of gut bacteria associated with social bees, with a curated genomic reference database. *Insect Science, 32*(1), 2-23. <https://doi.org/https://doi.org/10.1111/1744-7917.13365>
